# Moderate Reduction in Dietary Net Energy Level Enhances Intestinal Health in Tunchang Pigs via Gut Microbiota Modulation

**DOI:** 10.3390/ani15192836

**Published:** 2025-09-28

**Authors:** Xilong Yu, Hongzhi Wu, Haoliang Chai, Dexin Zhao, Weiqi Peng, Fengjie Ji, Lidong Zhang, Renlong Lv

**Affiliations:** 1 Tropical Crops Genetic Resources Research Institute, Chinese Academy of Tropical Agricultural Sciences, Haikou 571101, China; 2 College of Animal Science and Technology, Northeast Agricultural University, Harbin 150030, China; 3 Xinjin Bangde Technology Co., Ltd., Chengdu 610000, China

**Keywords:** Tunchang pig, low net-energy diet, gut microbiota, intestinal barrier function, short-chain fatty acids, intestinal health

## Abstract

To investigate the effects of low net-energy (NE) diets on intestinal health in Tunchang pigs, 96 animals (25.40 ± 1.11 kg) were randomly assigned to four dietary treatment groups with NE levels of 9.82 (CG), 9.57 (EY1), 9.32 (EY2), and 9.07 (EY3) MJ/kg. Each group consisted of six replicates with four pigs per replicate. The experiment lasted for 63 days. The results showed that compared with the CG, the EY2 increased jejunal villus height and villus height-to-crypt depth ratio, as well as reduced crypt depth in the colon (*p* < 0.05). Both the EY1 and EY2 demonstrated improved intestinal barrier function through upregulation of zonula occludens-1 and occludin expression in the jejunum, zonula occludens-1 in the ileum, and zonula occludens-1, occludin, and claudin-1 in the colon (*p* < 0.05). Furthermore, EY2 significantly increased the activities of superoxide dismutase, glutathione peroxidase, and catalase, while reducing malondialdehyde content in both the jejunum and colon (*p* < 0.05). EY2 showed significantly downregulated relative expression of pro-inflammatory cytokines, including interleukin-1β, tumor necrosis factor-α, and interleukin-6, in the jejunum, ileum, and colon (*p* < 0.05). Microbial and short-chain fatty acid (SCFA) analyses showed that the EY2 increased the abundance of beneficial bacteria such as *Faecalibacterium*, *CF231*, *Coprococcus*, *Ruminococcus*, and *Blautia* and elevated the concentrations of acetate, propionate, and butyrate. In summary, reducing dietary NE levels to no less than 9.32 MJ/kg improved intestinal health by modulating the gut microbiota and increasing SCFA production.

## Simple Summary

Energy constitutes a substantial portion of feed costs, and rising prices are driving producers to optimize feed management through precise dietary formulations. However, research on the effects of low net-energy diets on the intestinal health of finishing pigs remains limited, particularly with respect to the underlying regulatory mechanisms. This study investigated the effects of low net-energy diets on the intestinal health of Tunchang pigs. Compared with the control group, a diet with a net energy level of 9.32 MJ/kg increased jejunal villus height and the villus-height-to-crypt-depth ratio, while enhancing intestinal barrier function by upregulating the expression of tight junction protein genes. Additionally, the 9.32 MJ/kg diet improved intestinal antioxidant and anti-inflammatory functions by boosting the activity of antioxidant enzymes and reducing the expression of pro-inflammatory cytokine genes. Furthermore, this diet increased the abundance of beneficial bacteria and elevated short-chain fatty acid (SCFA) levels. In conclusion, a moderate reduction in dietary net energy (by no more than 0.5 MJ/kg) improved intestinal barrier function, antioxidant capacity, and anti-inflammatory response by modulating the gut microbiota and enhancing SCFA production. This study provides a theoretical basis for the application of low net-energy diets in finishing pig production.

## 1. Introduction

Pork, a dense reservoir of high-quality protein, lipids, essential amino acids, and minerals, constitutes a pivotal component of the human diet. Worldwide, it represents more than one-third of total meat consumed, while China alone is responsible for roughly half of global output and intake [1]. The vast scale of pig production in China places considerable pressure on feed resources, with corn and soybean meal serving as the primary components of commercial swine diets, supplying energy and protein, respectively. Although corn remains a primary staple crop in China, its supply has become increasingly constrained due to the growing demand for animal feed. China’s corn imports rose sharply from 11.3 million tons in 2020 to 28.35 million tons in 2021, with approximately 80% allocated to energy components in livestock feed production. Energy-rich feed typically accounts for about 70% of total swine feed costs [2], with corn serving as the principal source. As a result, the surge in global corn prices has further driven up pig production costs [3]. To address the challenges posed by supply shortages and escalating costs of key energy ingredients such as corn, exploring low-energy diets offers a promising avenue for reducing feed expenses.

The intestine plays vital roles in digestion, nutrient absorption, barrier defense, and immune function, making intestinal health indispensable for optimal animal growth and overall well-being [4]. This health is maintained through dynamic homeostasis arising from interactions among host factors such as tight junctions, intestinal epithelial cells, the mucosal layer and immune barriers, the gut environment including microbiota, and nutrients together with their metabolites, all of which are significantly influenced by dietary composition [5]. Previous research on diets with reduced energy levels has yielded inconsistent results, though the overall impact on intestinal health appears largely negative. For instance, low-energy diets have been reported to decrease villus height and increase crypt depth in weaned piglets, consequently compromising the integrity of the intestinal epithelial barrier [6]. Damage to the epithelial barrier can permit the translocation of toxins, allergens, or bacteria into systemic tissues, thereby triggering inflammatory and immune responses. In line with this, a 30% reduction in energy intake has been shown to downregulate the expression of genes associated with immunity and inflammation in the mouse colon [7]. Furthermore, low-energy diets have been shown to reduce microbial diversity and increase the abundance of the opportunistic pathogen *Pseudomonas* in the cecum of broilers [8]. However, some studies suggest that compensatory mechanisms may help maintain intestinal health under moderate energy restriction. For example, Li et al. [9] reported that a low-energy diet upregulated Junctional adhesion molecule-3 expression in the duodenum of rabbits while concurrently downregulating inflammatory factors, including Myeloid differentiation primary response protein 88 and interleukin-6 (*IL-6*), in the ileum. Moreover, a diet with a 5% reduction in both energy and protein was found to enhance intestinal antioxidant capacity in broilers [10]. Taken together, these findings highlight the need for further research to fully elucidate the effects of low-energy diets on animal intestinal health.

The Tunchang pig, a local breed from Hainan Province, China, is renowned for its high-quality meat, robust adaptability, and strong disease resistance. Despite its economic and agricultural importance, research on the effects of low net-energy (NE) diets on the intestinal health of finishing pigs remains limited, particularly with respect to the underlying regulatory mechanisms. Accordingly, this study aims to evaluate the impact of low-NE diets on intestinal morphology, antioxidant capacity, and inflammatory responses in finishing Tunchang pigs. Additionally, we evaluated the role of the gut microbiota and its metabolic products to gain insights into the potential underlying mechanisms.

## 2. Materials and Methods

### 2.1. Ethics Statement

The management and care procedures for the animals used in this study were approved by the Institutional Animal Care and Use Committee of the Chinese Academy of Tropical Agricultural Sciences (Approval No.: CATAS-20240916-2).

### 2.2. Animals, Diets, and Experimental Design

A total of 96 Tunchang pigs, 55 days of age and comprising females or castrated males, with an initial body weight of 25.40 ± 1.11 kg, were obtained from a farm in Bangxi Town, Baisha Li Autonomous County, Hainan Province, China. This study utilized a completely randomized design. The pigs were randomly assigned to four treatment groups, each with six replicates and four pigs per replicate. The four groups were fed corn–soybean meal diets with NE levels of 9.82, 9.57, 9.32, and 9.07 MJ/kg, designated as CG, EY1, EY2, and EY3, respectively (Table 1). The CG diet was formulated strictly according to the nutritional standards of Nutrient Requirements of Swine (GB/T 39235-2020) [11], while the other three treatment diets were identical in nutrient composition except for NE level. Pigs were housed in individual pens randomly distributed within the same barn, with pens cleaned daily, and had ad libitum access to feed and water throughout the 63-day feeding trial. Their feeding behavior, excretion patterns, and overall health were closely monitored. Daily feed offered and leftovers were recorded to calculate the average daily feed intake. On days 1 and 63, each pig was individually placed in a weighing crate and weighed using an electronic scale (Yongkang HUAYING Weighing Apparatus Co., Ltd., Yongkang, China) to record its initial and final body weight, respectively.

### 2.3. Sample Collection

At 63 days of age, following a 12 h fasting period, one Tunchang pig with body weight closest to the average from each replicate (4 pigs per replicate) was selected and slaughtered at a commercial slaughterhouse using electrical stunning and exsanguination. Approximately 2–3 cm tissue segments from the jejunum, ileum, and colon of each pig were collected and immediately fixed in 4% paraformaldehyde for intestinal morphological analysis. The remaining jejunal, ileal, and colonic tissues were stored at −80 °C for subsequent assessment of intestinal barrier function, antioxidant capacity, and inflammatory responses. Finally, colonic content samples were collected into 2 mL cryotubes, snap-frozen in liquid nitrogen, and preserved for microbial 16S rRNA gene sequencing and short-chain fatty acid analysis.

### 2.4. Intestinal Histomorphology

Following 24 h fixation in 4% paraformaldehyde, jejunal, ileal, and colonic segments were dehydrated through a graded ethanol series and embedded in paraffin. Serial sections (5 µm) were cut on a rotary microtome (RM2235, Leica Microsystems, Wetzlar, Germany) and stained with haematoxylin and eosin for histological assessment. Morphometric images were acquired on a DMi8 inverted microscope (Leica Microsystems) equipped with a digital camera, and villus height and crypt depth were quantified using ImageJ (version 2.16.0, National Institutes of Health, Bethesda, MD, USA).

### 2.5. Antioxidant Index Detection

Approximately 100 mg of frozen jejunal, ileal, and colonic tissue from Tunchang pigs was retrieved from −80 °C, rapidly thawed in ice-cold physiological saline, and homogenized (1:9, *w*/*v*) to yield a 10% (*w*/*v*) suspension. After centrifugation at 2500× *g* for 10 min at 4 °C, the clarified supernatant was collected. Activities of catalase (CAT), superoxide dismutase (SOD), and glutathione peroxidase (GSH-Px), as well as the concentration of malondialdehyde (MDA), were determined with commercial kits (Nanjing Jiancheng Bioengineering Institute, Nanjing, China) in strict accordance with the manufacturer’s specifications.

### 2.6. Determination of Specific Gene Expression Using Quantitative Real-Time PCR

Total RNA was extracted from jejunal, ileal, and colonic tissues using RNAiso Plus reagent (Takara, Shiga, Japan). The integrity and quality of the RNA were assessed by 2% agarose gel electrophoresis, while the concentration and purity were determined using a NanoDrop ND-2000 spectrophotometer (Thermo Fisher Scientific, Waltham, MA, USA). Then, 1.5 μg of total RNA was reverse-transcribed into first-strand complementary DNA (cDNA) using the PrimeScript^®^ RT Reagent Kit with gDNA Eraser (TaKaRa, Dalian, China). Quantitative real-time PCR (qRT-PCR) was performed on an Applied Biosystems 7500 Fast Real-Time PCR System (Foster City, CA, USA) to detect the expression levels of tight junction-related genes, including zona occludens-1 (*ZO-1*), claudin-1, and occludin, as well as intestinal inflammation-related genes ukin-1 beta (*IL-1β*), tumor necrosis factor alpha (*TNF-α*), and *IL-6*. β-Actin was used as the internal reference gene. Primer sequences are listed in Table S1. The relative mRNA expression levels of tight junction and inflammation-related genes were quantified using the 2^−ΔΔCt^ method, normalized to β-actin expression. Final gene expression values are reported as the mean relative expression normalized to the internal control.

### 2.7. Intestinal Microbiota Analysis

Microbial DNA was extracted from colonic digesta using a DNeasy PowerSoil kit (QIAGEN, Hilden, Germany). DNA concentration was determined with the Quant-iT PicoGreen dsDNA assay (Invitrogen, Carlsbad, CA, USA) on a Quantus fluorometer (Promega, Madison, WI, USA), and integrity was verified by 1% agarose gel electrophoresis. The V3–V4 hypervariable region of the 16S rRNA gene was amplified by PCR; amplicons were purified on 2% agarose gels and recovered with an AxyPrep DNA Gel Extraction Kit (Axygen, Union City, CA, USA). Purified amplicon concentrations were re-quantified using the same PicoGreen assay on a BioTek FL ×800 microplate reader (BioTek, Winooski, VT, USA). Libraries were constructed with the TruSeq Nano DNA LT kit (Illumina, San Diego, CA, USA) and sequenced (2 × 250 bp paired-end) on a NovaSeq 6000 using the SP reagent kit (Illumina).

Sequence data were processed in QIIME 2 (version 2019.4) following a modified version of the official tutorial. Raw reads were demultiplexed (demux plugin), primer sequences were trimmed with cutadapt, and quality control, denoising, merging, and chimera removal were performed with DADA2. Amplicon sequence variants (ASVs) were inferred at 100% identity, yielding an ASV table. Alpha-diversity indices were calculated, and beta diversity was assessed using weighted and unweighted UniFrac distances and visualized by principal-coordinate analysis (PCoA). Differentially abundant taxa between groups were identified with LEfSe (linear discriminant analysis effect size). Raw and processed reads have been deposited in the NCBI Sequence Read Archive under BioProject PRJNA1311709.

### 2.8. Determination of Short-Chain Fatty Acids in Colon Contents

An accurately weighed aliquot of colonic digesta was transferred into a 1.5 mL microcentrifuge tube and suspended in 500 µL of ultrapure water. After the addition of 100 mg acid-washed glass beads, the slurry was homogenized by vortexing for 1 min. Following centrifugation at 12,000× *g* and 4 °C for 10 min, 200 µL of the supernatant was combined with 100 µL of 15% (*v*/*v*) phosphoric acid, 20 µL of the internal standard (4-methylvaleric acid, 375 µg mL^−1^), and 280 µL of diethyl ether, then vigorously vortex-mixed for 1 min. The emulsion was re-centrifuged under identical conditions. The clear upper (organic) layer was recovered and analyzed for short-chain fatty acids (SCFAs) by gas chromatography-mass spectrometry (GC-MS) using a TRACE 1300 GC coupled to an ISQ 7000 mass spectrometer (Thermo Fisher Scientific, Waltham, MA, USA).

### 2.9. Statistical Analysis

Statistical analyses were conducted using SPSS software (version 26.0), and graphs were created with GraphPad Prism (version 8.0) for visualization. All statistical analyses utilized the replicate group as the experimental unit. Final body weight was calculated as the mean value of all pigs within each replicate group. For all other metrics, the measurement obtained from one pig per replicate, selected for having a body weight closest to the replicate mean, was used as the representative value for its entire replicate group. Before analysis, data were assessed for normality using the Shapiro–Wilk test and for homogeneity of variances using Levene’s test. Parametric data that met these assumptions were analyzed by one-way analysis of variance (ANOVA). When significant differences were detected, Duncan’s multiple range test was applied for post hoc comparisons. Results are presented as mean ± standard error of the mean (SEM), and differences were considered statistically significant at *p* < 0.05.

## 3. Results

### 3.1. Effects of Low Net-Energy Diets on the Growth Performance of Tunchang Pigs

**Figure 1:**
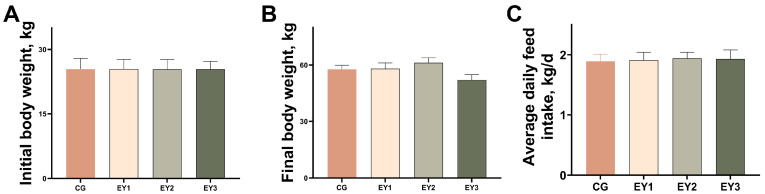
Effects of low net-energy diets on the growth performance of Tunchang pigs. (**A**) Initial body weight. (**B**) Final body weight. (**C**) Average daily feed intake. Data are presented as means ± SEM (*n* = 6). CG = the diet with a net energy level of 9.82 MJ/kg; EY1 = the diet with a net energy level of 9.57 MJ/kg; EY2 = the diet with a net energy level of 9.32 MJ/kg; EY3 = the diet with a net energy level of 9.07 MJ/kg.

As shown in Figure 1, the final body weight of Tunchang pigs was highest at a dietary NE level of 9.32 MJ/kg and lowest at 9.07 MJ/kg. However, there were no statistically significant differences in the final body weight among the groups (*p* > 0.05). Similarly, the average daily feed intake did not differ significantly among the treatment groups (*p* > 0.05).

### 3.2. Effects of Low Net-Energy Diets on the Intestinal Morphology of Tunchang Pigs

**Figure 2:**
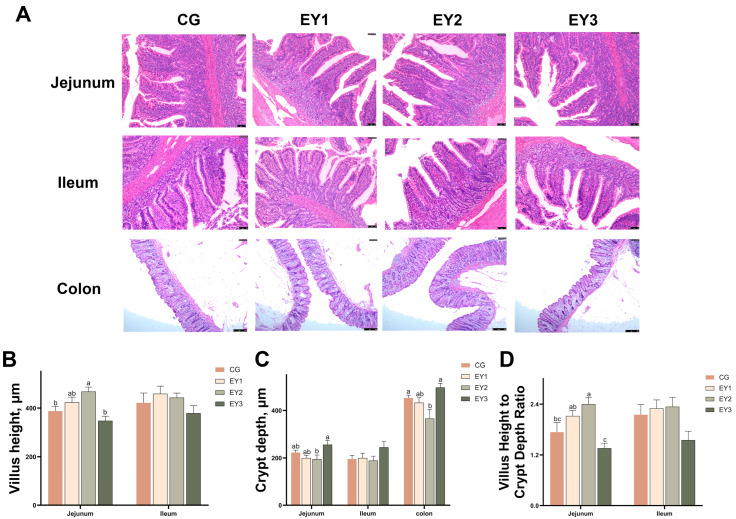
Effects of low net-energy diets on the intestinal morphology of Tunchang pigs. (**A**) Hematoxylin and eosin (H&E) staining of crypt and villus. (**B**) Villus height of the jejunum and ileum. (**C**) Crypt depths of the jejunum, ileum, and colon. (**D**) Villus height to crypt depth ratio in the jejunum and ileum. Data are presented as means ± SEM (*n* = 6); mean values with different letters indicate significant differences (*p* < 0.05). CG = the diet with a net energy level of 9.82 MJ/kg; EY1 = the diet with a net energy level of 9.57 MJ/kg; EY2 = the diet with a net energy level of 9.32 MJ/kg; EY3 = the diet with a net energy level of 9.07 MJ/kg.

Histological sections of Tunchang pig intestine (Figure 2A) revealed that, in the jejunum, pigs in the EY2 group possessed markedly taller villi and a higher villus-to-crypt (V/C) ratio than those in either the CG or EY3 groups (*p* < 0.05; Figure 2B,D). Concomitantly, jejunal crypt depth was reduced in EY2 pigs relative to the EY3 group (*p* < 0.05; Figure 2C). In the colon, crypt depth was greater in CG and EY3 pigs compared with EY2 counterparts (*p* < 0.05; Figure 2C). Within the ileum, villus height, crypt depth, and V/C ratio were unaffected by dietary NE level (*p* > 0.05; Figure 2B–D).

### 3.3. Effects of Low Net-Energy Diets on the Intestinal Antioxidant Capacity of Tunchang Pigs

**Figure 3:**
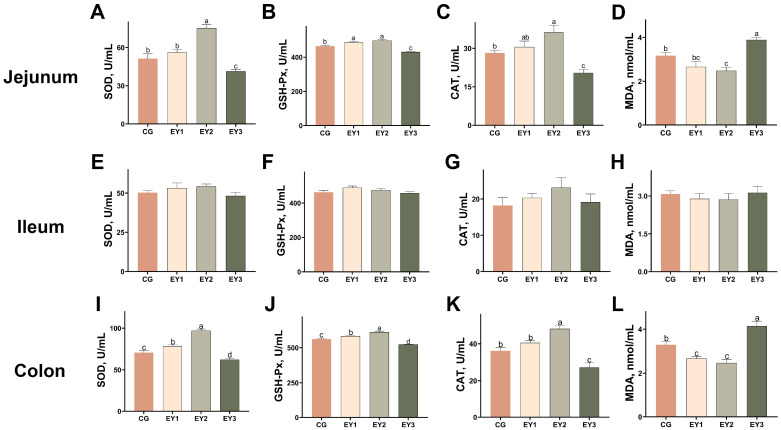
Effects of low net-energy diets on the intestinal antioxidant capacity of Tunchang pigs. Antioxidant indices, including SOD (**A**), GSH-Px (**B**), CAT (**C**), and MDA (**D**), in the jejunum tissue. Antioxidant indices, including SOD (**E**), GSH-Px (**F**), CAT (**G**), and MDA (**H**), in the ileum tissue. Antioxidant indices, including SOD (**I**), GSH-Px (**J**), CAT (**K**), and MDA (**L**), in the colon tissue. Data are presented as means ± SEM (*n* = 6); mean values with different letters indicate significant differences (*p* < 0.05). CG = the diet with a net energy level of 9.82 MJ/kg; EY1 = the diet with a net energy level of 9.57 MJ/kg; EY2 = the diet with a net energy level of 9.32 MJ/kg; EY3 = the diet with a net energy level of 9.07 MJ/kg.

Compared to the CG group, the EY2 diet enhanced the antioxidant status of Tunchang pigs by increasing SOD and CAT activities while reducing MDA concentration in the jejunum (*p* < 0.05; Figure 3A,C,D). These improvements were reversed under the EY3 diet, which suppressed both SOD and CAT activities and elevated MDA levels (*p* < 0.05). Jejunal GSH-Px activity was elevated in both EY1 and EY2 groups but decreased significantly with EY3 (*p* < 0.05; Figure 3B). Notably, no significant differences were observed in any measured indicators in the ileum among the groups (*p* > 0.05; Figure 3E–H). In the colon, both EY1 and EY2 diets improved antioxidant capacity, significantly increasing the activities of SOD and GSH-Px (*p* < 0.05; Figure 3I,J) and simultaneously suppressing the lipid peroxidation product MDA (*p* < 0.05; Figure 3L). In contrast, the EY3 regimen induced opposite effects, promoting MDA accumulation (*p* < 0.05) while reducing the activities of SOD and GSH-Px (*p* < 0.05). CAT activity followed a similar pattern, being up-regulated in the EY2 group compared to CG but markedly down-regulated in EY3 (*p* < 0.05; Figure 3K).

### 3.4. Effects of Low Net-Energy Diets on the Intestinal Barrier of Tunchang Pigs

**Figure 4:**
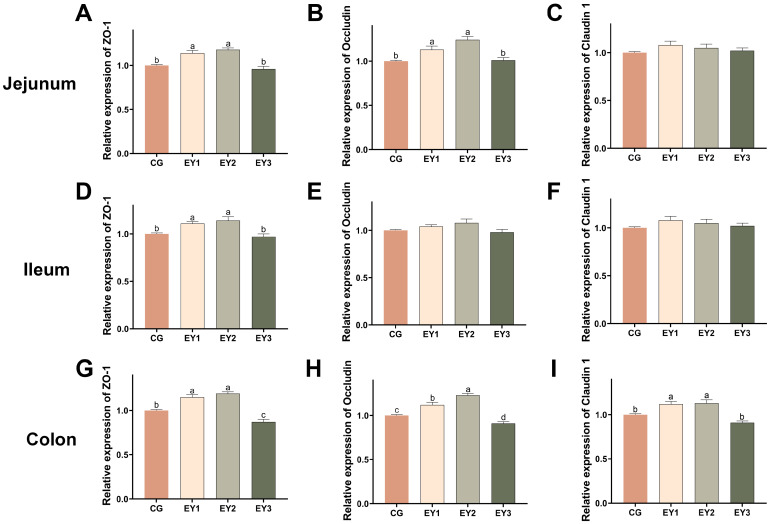
Effects of low net-energy diets on the intestinal barrier of Tunchang pigs. mRNA relative expression levels of tight junction proteins *ZO-1* (**A**), occludin (**B**), and claudin-1 (**C**) in the jejunum. mRNA relative expression levels of tight junction proteins *ZO-1* (**D**), occludin (**E**), and claudin-1 (**F**) in the ileum. mRNA relative expression levels of tight junction proteins *ZO-1* (**G**), occludin (**H**), and claudin-1 (**I**) in the colon. Data are presented as means ± SEM (*n* = 6); mean values with different letters indicate significant differences (*p* < 0.05). CG = the diet with a net energy level of 9.82 MJ/kg; EY1 = the diet with a net energy level of 9.57 MJ/kg; EY2 = the diet with a net energy level of 9.32 MJ/kg; EY3 = the diet with a net energy level of 9.07 MJ/kg.

Relative to CG, jejunal, ileal, and colonic *ZO-1* mRNA abundance rose markedly in EY1 and EY2 (*p* < 0.05, Figure 4A,D,G), whereas it declined only in the colonic mucosa of EY3 (*p* < 0.05, Figure 4G). A comparable pattern emerged for occludin jejunal and colonic expression, which was significantly amplified in EY1 and EY2 (*p* < 0.05, Figure 4B,H) yet diminished in the colon of EY3 (*p* < 0.05, Figure 4H). Occludin abundance in the ileum remained unchanged across treatments (*p* > 0.05, Figure 4E). Claudin-1 colonic transcripts were also markedly up-regulated in EY1 and EY2 (*p* < 0.05, Figure 4I), while jejunal and ileal levels did not differ from CG (*p* > 0.05, Figure 4C,F).

### 3.5. Effects of Low Net-Energy Diets on the Intestinal Inflammatory Response of Tunchang Pigs

**Figure 5:**
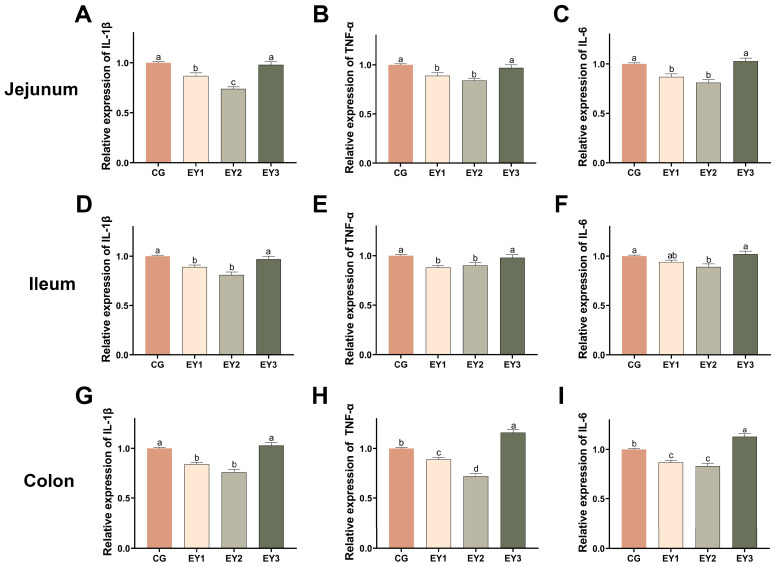
Effects of low net-energy diets on the intestinal inflammatory response of Tunchang pigs. mRNA relative expression levels of pro-inflammatory factors *IL-1β* (**A**), *TNF-α* (**B**), and *IL-6* (**C**) in the jejunum. mRNA relative expression levels of pro-inflammatory factors *IL-1β* (**D**), *TNF-α* (**E**), and *IL-6* (**F**) in the ileum. mRNA relative expression levels of pro-inflammatory factors *IL-1β* (**G**), *TNF-α* (**H**), and *IL-6* (**I**) in the colon. Data are presented as means ± SEM (*n* = 6); mean values with different letters indicate significant differences (*p* < 0.05). CG = the diet with a net energy level of 9.82 MJ/kg; EY1 = the diet with a net energy level of 9.57 MJ/kg; EY2 = the diet with a net energy level of 9.32 MJ/kg; EY3 = the diet with a net energy level of 9.07 MJ/kg.

Relative jejunal and ileal mRNA abundances of *IL-1β* and *TNF-α* were markedly lower in both EY1 and EY2 pigs than in controls (*p* < 0.05, Figure 5A,B,D,E). A parallel reduction in jejunal *IL-6* was evident in EY1 and EY2 (*p* < 0.05, Figure 5C), whereas in the ileum, a significant decrease in *IL-6* appeared only in EY2 (*p* < 0.05, Figure 5F). In the colon, EY1 and EY2 again displayed lower expression of *IL-1β*, *TNF-α*, and *IL-6* relative to CG (*p* < 0.05, Figure 5G–I). Conversely, EY3 exhibited elevated colonic *TNF-α* and *IL-6* compared with CG (*p* < 0.05, Figure 5H,I).

### 3.6. Diversity Changes in Gut Microbiota Under Low Net-Energy Diets

**Figure 6:**
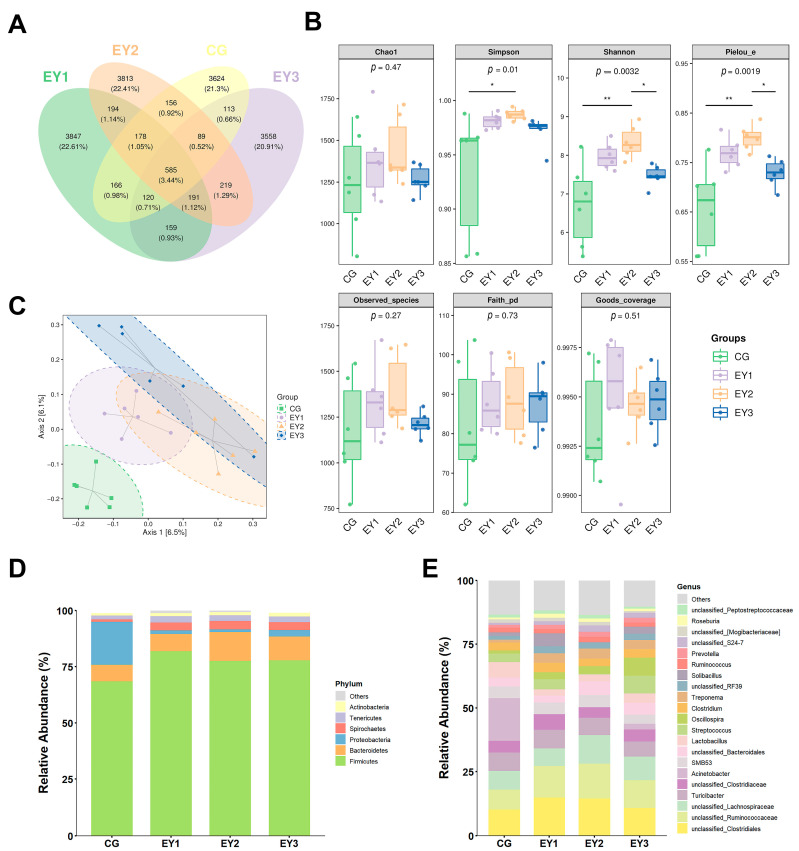
Effects of low net-energy diets on the colonic microbial composition of Tunchang pigs (*n* = 6). (**A**) Venn diagram of colonic microorganisms in each treatment group. (**B**) Colonic microbial alpha diversity. (**C**) Colonic microbial beta diversity. Key colon microbiota at the phylum (**D**) and genus (**E**) levels. * and ** denote significant differences at *p* < 0.05 and *p* < 0.01, respectively. CG = the diet with a net energy level of 9.82 MJ/kg; EY1 = the diet with a net energy level of 9.57 MJ/kg; EY2 = the diet with a net energy level of 9.32 MJ/kg; EY3 = the diet with a net energy level of 9.07 MJ/kg.

Analysis of 16S rRNA sequencing data revealed the impact of low-NE diets on the colonic microbiota of Tunchang pigs. A total of 17,012 ASVs were identified across the CG, EY1, EY2, and EY3 groups, with 585 core ASVs shared among all groups (Figure 6A). Rarefaction curves plateaued progressively (Figure S1A–C), confirming sufficient sequencing depth to characterize microbial diversity and community composition. Compared to CG, the EY1, EY2, and EY3 groups exhibited elevated α-diversity indices (Chao1, Simpson, Shannon, Pielou_e, Observed_species, Faith_pd, and Goods_coverage), though only EY2 showed statistically significant increases in Simpson, Shannon, and Pielou_e indices (Figure 6B, *p* < 0.05).

### 3.7. Compositional Changes in Gut Microbiota Under Low Net-Energy Diets

The PCoA based on Jaccard distance showed a clear separation between the CG group and the other groups, indicating that a reduction in the dietary NE level induced significant differences in the microbiota composition (Figure 6C). The PERMANOVA statistical test reinforced this observation, confirming the differences between the CG and experimental groups (*p* = 0.001, Figure S1D). By assessing the impact of low-NE diets on the bacterial representation across different taxa, it was found that the major phyla in each treatment group were *Firmicutes*, *Bacteroidetes*, *Proteobacteria*, *Spirochaetes*, and *Tenericutes*. Among them, *Firmicutes* dominated (accounting for over 65%), with its abundance being lower in the CG group compared to the experimental groups (Figure 6D). In addition, compared to the CG group, the samples from EY1, EY2, and EY3 exhibited higher abundances of *Bacteroidetes*, *Spirochaetes*, and *Tenericutes*, while the abundance of *Proteobacteria* decreased. At the genus level, *Acinetobacter* dominated in the CG group, but its abundance was significantly reduced in the experimental groups. Furthermore, the abundance of *Turicibacter* and *SMB53* was slightly decreased in the EY3 group. In comparison to CG, the experimental groups showed an increase in the abundance of *unclassified_Ruminococcaceae*, *unclassified_Clostridiales*, *Treponema*, and *Oscillospira*, while *Lactobacillus* abundance decreased. The abundance of *Streptococcus* and *Solibacillus* decreased in the EY2 group but increased in the EY1 and EY3 groups (Figure 6E).

### 3.8. Impact of Low Net-Energy Diets on Gut Microbiota via LEfSe Analysis

**Figure 7:**
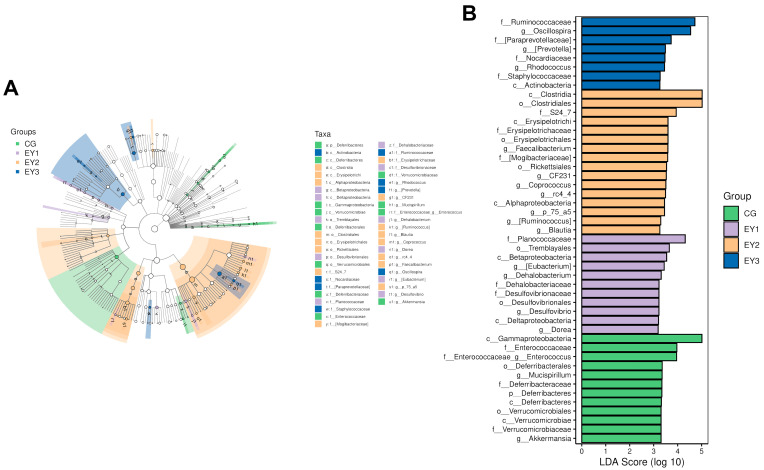
Effects of low net-energy diets on the colonic microbial composition of Tunchang pigs (*n* = 6). (**A**) Phylogenetic tree derived from LEfSe analysis. (**B**) LDA histograms. CG = the diet with a net energy level of 9.82 MJ/kg; EY1 = the diet with a net energy level of 9.57 MJ/kg; EY2 = the diet with a net energy level of 9.32 MJ/kg; EY3 = the diet with a net energy level of 9.07 MJ/kg.

LEfSe analysis employing Linear Discriminant Analysis (LDA) identified differentially abundant bacterial taxa and classified differential bacteria (Figure 7A,B). Under thresholds of *p* < 0.05 and LDA > 3, 47 significantly enriched taxonomic features were detected from phylum to species level. At the phylum level, only *Deferribacteres* was significantly enriched in the CG. Genus-level analysis revealed significant enrichment of *Mucispirillum*, *Akkermansia*, and *Enterococcus* in CG (*p* < 0.05), while *Eubacterium*, *Dehalobacterium*, *Desulfovibrio*, and *Dorea* were enriched in EY1 (*p* < 0.05). The EY2 group exhibited enrichment of *Faecalibacterium*, *CF231*, *Coprococcus*, *rc4_4*, *p_75_a5*, *Ruminococcus*, and *Blautia* (*p* < 0.05). Notably, *Oscillospira*, *Prevotella*, and *Rhodococcus* were significantly enriched in EY3 (*p* < 0.05).

### 3.9. Changes in Gut Short-Chain Fatty Acids

**Figure 8:**
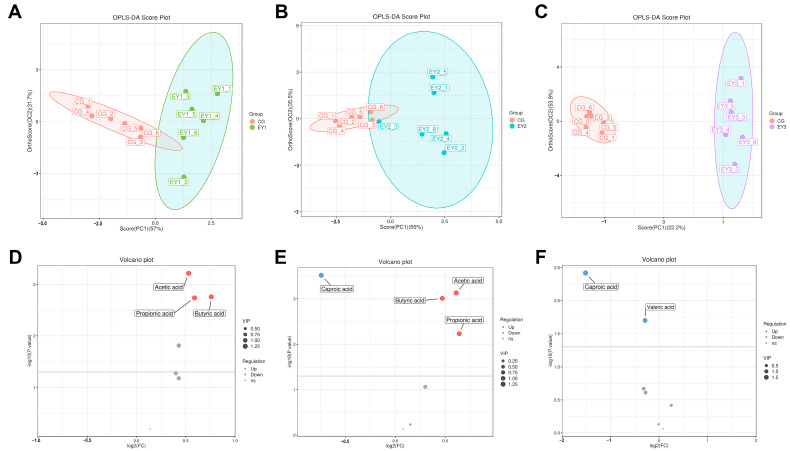
Effects of a low net-energy diet on colonic short-chain fatty acid composition in Tunchang pigs (*n* = 6). (**A**–**C**) Orthogonal partial least squares-discriminant analysis (OPLS-DA) score plots of short-chain fatty acids between the CG and EY1, EY2, and EY3 groups. (**D**–**F**) Volcano plot of differential short-chain fatty acids between CG and EY1, EY2, and EY3 groups. CG = the diet with a net energy level of 9.82 MJ/kg; EY1 = the diet with a net energy level of 9.57 MJ/kg; EY2 = the diet with a net energy level of 9.32 MJ/kg; EY3 = the diet with a net energy level of 9.07 MJ/kg.

Quality control analysis of the data showed that the relative standard deviation (RSD) was below 10%, indicating good data quality (Figure S2B). Hierarchical clustering analysis of all samples initially grouped the CG into a small cluster (Figure S2A). As the Euclidean distance increased, the CG cluster gradually merged with the EY3, EY2, and EY1 groups, eventually forming a larger cluster. This suggests differences in SCFA composition between the experimental groups and the CG. The OPLS-DA analysis further confirmed this finding (Figure 8A–C), showing clear separation between the CG and experimental groups along the first principal component.

Differential SCFAs between EY1, EY2, EY3 groups and the CG were identified using t-tests (*p* < 0.05) combined with variable importance in projection (VIP > 1), and visualized by volcano plots (Figure 8D–F). The results showed that three SCFAs (Acetic acid, Propionic acid, and Butyric acid) were upregulated in EY1 compared to CG (*p* < 0.05). In EY2, four SCFAs differed significantly from CG, with three upregulated (Acetic acid, Propionic acid, and Butyric acid) and one downregulated (Caproic acid) (*p* < 0.05). Additionally, compared to CG, the EY3 group exhibited significant decreases in Caproic acid and Valeric acid (*p* < 0.05).

### 3.10. Correlation Analysis Between Differentially Abundant Microbial Genera and Short-Chain Fatty Acids

**Figure 9:**
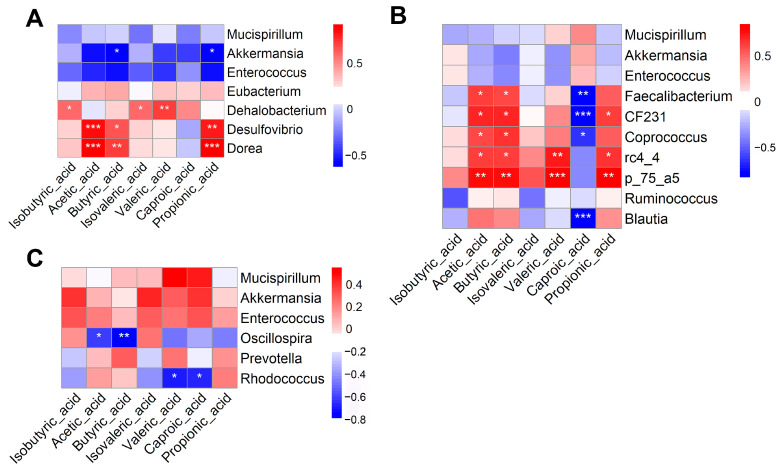
Pearson correlation analysis between key bacterial genera and short-chain fatty acids (*n* = 6). (**A**–**C**) Pearson Correlation Analysis between CG and EY1, EY2, EY3 groups. CG = the diet with a net energy level of 9.82 MJ/kg; EY1 = the diet with a net energy level of 9.57 MJ/kg; EY2 = the diet with a net energy level of 9.32 MJ/kg; EY3 = the diet with a net energy level of 9.07 MJ/kg. Asterisks indicate the significance level of Pearson correlation coefficients between gut microbiota genera and short-chain fatty acids (* *p* < 0.05, ** *p* < 0.01, *** *p* < 0.001).

The Pearson correlation analysis revealed significant associations between key bacterial genera and SCFAs. In the comparison between the EY1 and CG groups (Figure 9A), acetic acid, propionic acid, and butyric acid were positively correlated with *Desulfovibrio* and *Dorea* (*p* < 0.05) and for EY2 vs. CG (Figure 9B), both acetic acid and butyric acid correlated positively with *Faecalibacterium*, *CF231*, *Coprococcus*, *rc4_4*, and *p_75_a5* (*p* < 0.05). In contrast, propionic acid exhibited positive correlations with *CF231*, *rc4_4*, and *p_75_a5* (*p* < 0.05). Conversely, caproic acid demonstrated significant negative correlations with *Faecalibacterium*, *CF231*, *Coprococcus*, and *Blautia* (*p* < 0.05). In the comparison between the EY3 and CG groups (Figure 9C), both caproic acid and valeric acid were negatively correlated with *Rhodococcus* (*p* < 0.05).

## 4. Discussion

The intestine plays a pivotal role in nutrient digestion and absorption, directly influencing animal growth performance [12]. Villus height determines the effective absorptive surface area [13], while the V/C ratio provides a direct measure of absorptive capacity, with higher ratios indicating a larger surface area and improved nutrient absorption efficiency [14]. Tight junctions, comprising transmembrane adhesion molecules such as claudin and occludin, along with the peripheral protein *ZO-1*, preserve mucosal barrier integrity and selectively regulate the transport of macromolecules, fluids, and immune cells [15]. In this study, lowering dietary NE to 9.32 MJ/kg increased jejunal villus height and the V/C ratio, while also upregulating the relative expression of *ZO-1* and occludin in the jejunum, *ZO-1* in the ileum, and *ZO-1*, occludin, and claudin-1 in the colon. These enhancements in intestinal morphology and barrier function may improve nutrient absorption [16], thereby supporting growth performance. However, when the dietary NE was further reduced to 9.07 MJ/kg, villus height and the V/C ratio declined, and the relative expression of *ZO-1* and occludin in the colon was significantly decreased, potentially compromising intestinal function and leading to reduced growth performance. Existing research shows inconsistent effects of energy level on gut morphology and barrier function, with weaned piglet studies indicating that energy intake determines villus height [17]. In contrast, broiler research reports no significant impact of low-energy diets on villus width, height, crypt depth, surface area, V/C ratio, or barrier-related genes (*claudin-1* and *ZO-1*) [18,19]. Moreover, Honghe Yellow cattle exhibited increased ruminal claudin-1 expression under low energy diets [20]. These discrepancies may arise from species differences, variations in the degree of energy reduction, or differences in feed composition. Notably, the gut microbiota may play a key role in mediating the effects of dietary energy on intestinal morphology and barrier function, acting as an interface between diet and host.

Key factors compromising intestinal barrier function are oxidative stress and inflammation [21]. Oxidative stress arises from an imbalance between oxidants and antioxidants, in which elevated levels of reactive oxygen species (ROS) or inadequate antioxidant defenses lead to oxidative damage [22]. Antioxidant enzymes play a critical role in mitigating oxidative stress and neutralizing ROS [23]. In particular, SOD catalyzes the dismutation of superoxide radicals into oxygen and hydrogen peroxide [24], while CAT breaks down hydrogen peroxide into water and oxygen [25]. Reduced activity of these enzymes diminishes overall antioxidant capacity, which leads to rapid oxidation of polyunsaturated fatty acids and accumulation of harmful substances such as ROS and MDA, ultimately causing intestinal oxidative damage [26,27]. Research on the effects of dietary energy levels on intestinal antioxidant capacity in fattening pigs remains limited, and findings across species are inconsistent. For instance, in poultry, low-nutrient-density diets, characterized by a reduction of 81 kcal in metabolizable energy and 0.43% in crude protein, significantly decreased jejunal mucosal T-AOC and total SOD activity [28]. However, studies on male Cobb 500 broilers have shown that low-energy diets enhance the overall intestinal antioxidant response [10]. In the present study, reducing dietary NE to 9.32 MJ/kg increased jejunal and colonic SOD, GSH-Px, and CAT activities, accompanied by a decrease in MDA levels. However, a further reduction in NE to 9.07 MJ/kg resulted in significant declines in SOD, GSH-Px, and CAT activities and a marked increase in MDA. It is well established that oxidative damage can activate inflammation-related transcription factors, including NF-κB, which subsequently induce the expression of pro-inflammatory cytokines (*IL-1β*, *IL-6*) and chemokines (*IL-8*) [29]. Correspondingly, before reaching 9.32 MJ/kg NE, the expression of pro-inflammatory factors (*IL-1β*, *TNF-α*, *IL-6*) in the jejunum, ileum, and colon decreased progressively, reaching their lowest levels at this energy concentration. These findings align with studies in rabbits, in which low digestible energy diets downregulated *IL-6* expression in the ileum [9]. Further reduction to 9.07 MJ/kg NE, however, led to upregulation of these inflammatory mediators, showing a trend opposite to that of antioxidant markers in the jejunum and colon, which may be attributed to excessive energy restriction. Overall, mild reductions in dietary NE of approximately 0.5 MJ/kg enhanced intestinal antioxidant capacity and anti-inflammatory responses, while further reductions to 9.07 MJ/kg impaired antioxidant defenses and worsened intestinal inflammation.

The gut microbiota plays a crucial role in intestinal stem cell-mediated epithelial renewal and maintaining mucosal barrier integrity [30]. Diet is a major factor shaping the gut microbiota. Previous studies on low-energy diets have demonstrated that reducing digestible energy by 150 kcal/kg did not alter the populations of *Escherichia coli*, *Lactobacillus*, total anaerobes, or total aerobes in the colon, cecum, and rectum of weaned piglets [31]. However, excessive energy restriction may reduce microbial diversity and alter the composition of the microbiota [32]. In this study, reducing the NE level by 0.5 MJ/kg improved the composition of the gut microbiota and increased microbial diversity. Specifically, the low-NE diet reduced the abundance of pathogenic bacteria, including *Mucispirillum* and *Enterococcus*, which were enriched in the CG. When the diet’s NE level was reduced to 9.57 MJ/kg, the abundance of beneficial bacteria, including *Eubacterium*, *Dehalobacterium*, and *Dorea* increased in the colon, and a further reduction to 9.32 MJ/kg led to a significant rise in beneficial bacteria such as *Faecalibacterium*, *CF231*, *Coprococcus*, *Ruminococcus*, and *Blautia*. *Mucispirillum* and *Enterococcus* are linked to intestinal inflammation, with their abundance positively correlating with inflammatory and oxidative stress markers [33,34]. *Eubacterium* enhances host antioxidant capacity through theaflavin metabolism, alleviates colitis, and promotes villus development [35,36]. *Dorea* significantly lowers the risk of inflammatory bowel disease [37]. *Faecalibacterium* inhibits inflammatory factor release and repairs epithelial barrier damage by upregulating occludin [38], while also correlating with elevated antioxidant gene expression in broilers [26]. *Coprococcus* and *Ruminococcus* help regulate intestinal inflammation; *Coprococcus* reduces pro-inflammatory cytokines and strengthens tight junction protein expression [39,40]. *Blautia* is also associated with mitigating intestinal inflammation and improving antioxidant capacity [41,42]. These changes in the gut microbiota may explain how reducing the NE level to 9.32 MJ/kg enhanced villus height, improved intestinal barrier function, and increased antioxidant capacity, while simultaneously lowering the expression of pro-inflammatory cytokines. However, when the NE level was further reduced to 9.07 MJ/kg, the abundance of pathogenic bacteria, including *Prevotella* and *Rhodococcus*, increased. *Prevotella* has been shown to promote the generation of Th17 cells, which leads to increased levels of pro-inflammatory factors including *IL-6*, *TNF-α*, and *IL-1β* [43]. *Rhodococcus* is known to induce colitis [44]. This phenomenon may explain the decreased antioxidant capacity, the reduced expression of tight junction proteins, and the increased expression of pro-inflammatory factors observed when the NE level dropped to 9.07 MJ/kg. In summary, reducing the NE level beyond a certain point may result in intestinal damage.

The gut microbiota supports host health through its composition and metabolites, while the host’s nutritional status, in turn, shapes both the microbial community and its metabolic outputs. Based on the correlation analysis between SCFAs and gut microbiota, and informed by functional insights into specific bacterial taxa reported in previous studies, it was observed that reducing the dietary NE level by 0.25 MJ/kg increased the abundance of *Desulfovibrio* in the gut of Tunchang pigs, thereby elevating acetic and propionic acid levels [45,46], and also promoted the abundance of *Dorea*, resulting in higher butyric acid levels [47]. When the NE level was reduced by 0.5 MJ/kg, the diet increased the abundance of *Faecalibacterium* [48,49] and *Coprococcus* [39,50], thereby elevating acetic and butyric acid concentrations. Additionally, propionic acid content was increased through a higher abundance of *CF231* [51], while the rise in *Blautia* abundance may have contributed to a decrease in caproic acid levels [52]. However, when the NE level was further reduced to 9.07 MJ/kg, the abundance of *Rhodococcus* in the gut increased, which corresponded with decreased levels of valeric and caproic acids, potentially reflecting *Rhodococcus’s* efficient capacity to biodegrade these acids [53]. These findings indicate that a moderate reduction in NE intake may enable the host to partially offset energy deficiency by enhancing the abundance of beneficial gut microbes and their metabolites, including acetate, butyrate, and propionate, which aligns with the classical concept that SCFAs can provide up to 25% of the energy requirements in monogastric animals [54]. However, when NE is further reduced to 9.07 MJ/kg, this compensatory mechanism becomes insufficient, resulting in aggravated energy deficiency, disruption of gut microbial balance, and a decline in SCFA production. SCFAs are essential for reinforcing the intestinal barrier, modulating mucosal immune responses, and alleviating oxidative stress [55]. Acetic acid enhances piglet immunity by activating innate and adaptive immune cells and strengthening intestinal barrier function via the PI3K-AKT pathway [56]. Propionic acid reduces intestinal inflammation by suppressing cytokines such as *IL-1β*, *CXCL8*, *IL-17*, and *IL-22* [57], and may enhance gut barrier integrity by increasing *FABP1*, occludin, and *ZO-1* gene expression [58]. Butyric acid, as the primary energy source for colonocytes, promotes the proliferation of colonic epithelial cells at low concentrations [59], supports the maintenance of barrier integrity, and exhibits strong anti-inflammatory and antioxidant properties [60]. Both caproic acid and valeric acid have been shown to inhibit *E. coli* colonization, support intestinal barrier repair, and strengthen immune function [61,62]. Therefore, in this study, reducing dietary NE by no more than 0.5 MJ/kg enhanced intestinal health by modulating the gut microbiota composition, which in turn increased SCFA levels. However, when the NE level was further reduced to 9.07 MJ/kg, the resulting alterations in the gut microbiota of Tunchang pigs led to decreased valeric and caproic acid concentrations, thereby compromising intestinal health. These changes in SCFAs may provide further insight into the mechanisms by which the gut microbiota affects intestinal function.

## 5. Conclusions

In conclusion, a moderate reduction in dietary NE by 0.5 MJ/kg improved intestinal health in Tunchang pigs by increasing the abundance of beneficial gut bacteria and enhancing SCFA production. This modulation of the gut microbiota led to improved barrier function, strengthened antioxidant capacity, and reduced inflammatory responses. In contrast, a further reduction in NE to 9.07 MJ/kg caused gut dysbiosis and a marked decrease in SCFA levels, ultimately impairing intestinal health.

## Figures and Tables

**Table 1:** Composition and Nutrient components for diets (air-dried basis, %).

Items ^1^	CG	EY1	EY2	EY3
Ingredients				
Corn	52.85	54.4	55.93	57.47
Soybean meal	9.08	8.75	8.43	8.10
Wheat bra	32.10	32.10	32.10	32.10
Soybean oil	3.64	2.42	1.21	0
Stone powder	1.30	1.30	1.30	1.30
NaCl	0.20	0.20	0.20	0.20
L-Lysin (98%)	0.03	0.03	0.03	0.03
Compound premix ^2^	0.50	0.50	0.50	0.50
Mold inhibitor	0.30	0.30	0.30	0.30
Total	100.00	100.00	100.00	100.00
Nutrition levels ^3^				
Net energy levels (MJ/kg)	9.82	9.57	9.32	9.07
Crude protein	13.52	13.52	13.52	13.52
Crude fiber	3.74	3.77	3.8	3.84
Crude fat	6.94	5.80	4.67	3.53
Ash	2.79	2.79	2.78	2.77
Calcium	0.53	0.53	0.53	0.53
Phosphorus	0.54	0.55	0.55	0.55
Lysine	0.68	0.68	0.68	0.68

^1^ CG, the diet with a net energy level of 9.82 MJ/kg; EY1, the diet with a net energy level of 9.57 MJ/kg; EY2, the diet with a net energy level of 9.32 MJ/kg; EY3, the diet with a net energy level of 9.07 MJ/kg. ^2^ The premix provided the following amounts of vitamins and trace elements per kg of diet: vitamin A, 5500 IU; vitamin D3, 2300 IU; vitamin E, 30 IU; vitamin K3, 2.2 mg; vitamin B6, 3 mg; vitamin B12, 27.6 μg; riboflavin, 4 mg; pantothenic acid, 14 mg; niacin, 30 mg; choline chloride, 400 mg; folacin, 0.7 mg; biotin, 44 μg; Fe (FeSO_4_), 90 mg; Mn (MnSO_4_), 40 mg; Zn (ZnO), 75 mg; Cu (CuSO_4_), 100 mg; I (Ca(IO_3_)_2_), 0.3 mg; Se (Na_2_SeO_3_), 0.3 mg. ^3^ Net energy and lysine levels are calculated values, and other nutrient levels are measured values.

## Data Availability

Data presented are original and not inappropriately selected, manipulated, enhanced, or fabricated. Raw and processed reads about 16S rDNA have been deposited in the NCBI Sequence Read Archive under BioProject PRJNA1311709.

## Supplementary Materials

The following supporting information can be downloaded at: https://www.mdpi.com/article/10.3390/ani15192836/s1, Table S1: The information of specific primer sequences used in qRT-PCR, Figure S1: Microbial diversity and community differences, Figure S2: Data quality control and hierarchical cluster analysis.
