# Supplementary material for: Moderate Reduction in Dietary Net Energy Level Enhances Intestinal Health in Tunchang Pigs via Gut Microbiota Modulation
Source: Animals (Basel). 2025 Sep 28;15(19):2836. doi: 10.3390/ani15192836 (PMC12523503; doi:10.3390/ani15192836)
Supplement: Supplementary file 1 [file animals-15-02836-s001.zip › animals-3882720-supplementary.pdf]

Table S1. Composition and Nutrient components for diets (air-dried basis, %).

Items[1]	CG	EY1	EY2	EY3
Ingredients				
Corn	52.85	54.4	55.93	57.47
Soybean meal	9.08	8.75	8.43	8.10
Wheat bra	32.10	32.10	32.10	32.10
Soybean oil	3.64	2.42	1.21	0
Stone powder	1.30	1.30	1.30	1.30
NaCl	0.20	0.20	0.20	0.20
L-Lysin (98%)	0.03	0.03	0.03	0.03
Compound premix[2]	0.50	0.50	0.50	0.50
Mold inhibitor	0.30	0.30	0.30	0.30
Total	100.00	100.00	100.00	100.00
Nutrition levels[3]				
Net energy levels (MJ/kg)	9.82	9.57	9.32	9.07
Crude protein	13.52	13.52	13.52	13.52
Crude fiber	3.74	3.77	3.8	3.84
Crude fat	6.94	5.80	4.67	3.53
Ash	2.79	2.79	2.78	2.77
Calcium	0.53	0.53	0.53	0.53
Phosphorus	0.54	0.55	0.55	0.55
Lysine	0.68	0.68	0.68	0.68

[1] CG, the diet with a net energy level of 9.82 MJ/kg; EY1, the diet with a net energy level of 9.57 MJ/kg; EY2, the diet with a net energy level of 9.32 MJ/kg; EY3, the diet with a net energy level of 9.07 MJ/kg.
[2] The premix provided the following amounts of vitamins and trace elements per kg of diet: vitamin A, 5,500 IU; vitamin D3, 2,300 IU; vitamin E, 30 IU; vitamin K3, 2.2 mg; vitamin B6, 3 mg; vitamin B12, 27.6 μg; riboflavin, 4 mg; pantothenic acid, 14 mg; niacin, 30 mg; choline chloride, 400 mg; folacin, 0.7 mg; biotin, 44 μg; Fe (FeSO4), 90 mg; Mn (MnSO4), 40 mg; Zn (ZnO), 75 mg; Cu (CuSO4), 100 mg; I ($Ca(IO_3)_2$), 0.3 mg; Se (Na_2SeO_3), 0.3 mg.
[3] Net energy and lysine levels are calculated values, and other nutrient levels are measured values.

Table S2. The information of specific primer sequences used in qRT-PCR.

Gene Names	Sequence (5' → 3')	NCBI Reference Sequence	Amplicon size (bp)
β-actin	GACGATATTGCTGCGCTCGT<br>TAGGAGTCCTTCTGGCCCAT	XM_021086047.1	152
ZO-1	ACTTGTCAGCTCAGCCAGTC<br>ACAGGCCTCAGAAATCCAGC	XM_021098896.1	82
Claudin-1	CGTAGCATCCTGGAGCAGTC<br>AGTCTGTGCCAATTGAGGCT	XM_005670262.3	122
Occludin	CTCGTCCAACGGGAAAGTGA<br>ACGCCTCCAAGTTACCACTG	NM_001163647.2	155
IL-1β	ACCTGGACCTTGGTTCTC<br>GGATTCTTCATCGGCTTC	XM_021085847.1	124
TNF-α	TAAGGGCTGCCTTGGTTCAG<br>AGAGGTTCAGCGATGTAGCG	X57321.1	187
IL-6	CGAGCCCACCAGGAACGAAAG<br>GCAGTAGCCATCACCAGAAGCAG	NM_214399.1	126

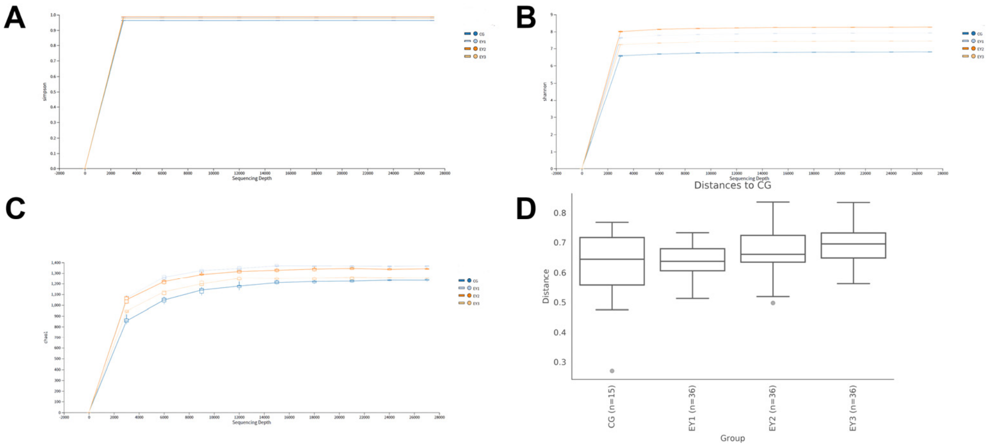

Figure S1. Microbial diversity and community differences ($n = 6$). (**A**) Rarefaction curve of the Simpson index. (**B**) Rarefaction curve of the Shannon index. (**C**) Rarefaction curve of the Chao1 index. (**D**) PERMANOVA statistical test. CG = the diet with a net energy level of 9.82 MJ/kg; EY1 = the diet with a net energy level of 9.57 MJ/kg; EY2 = the diet with a net energy level of 9.32 MJ/kg; EY3 = the diet with a net energy level of 9.07 MJ/kg.

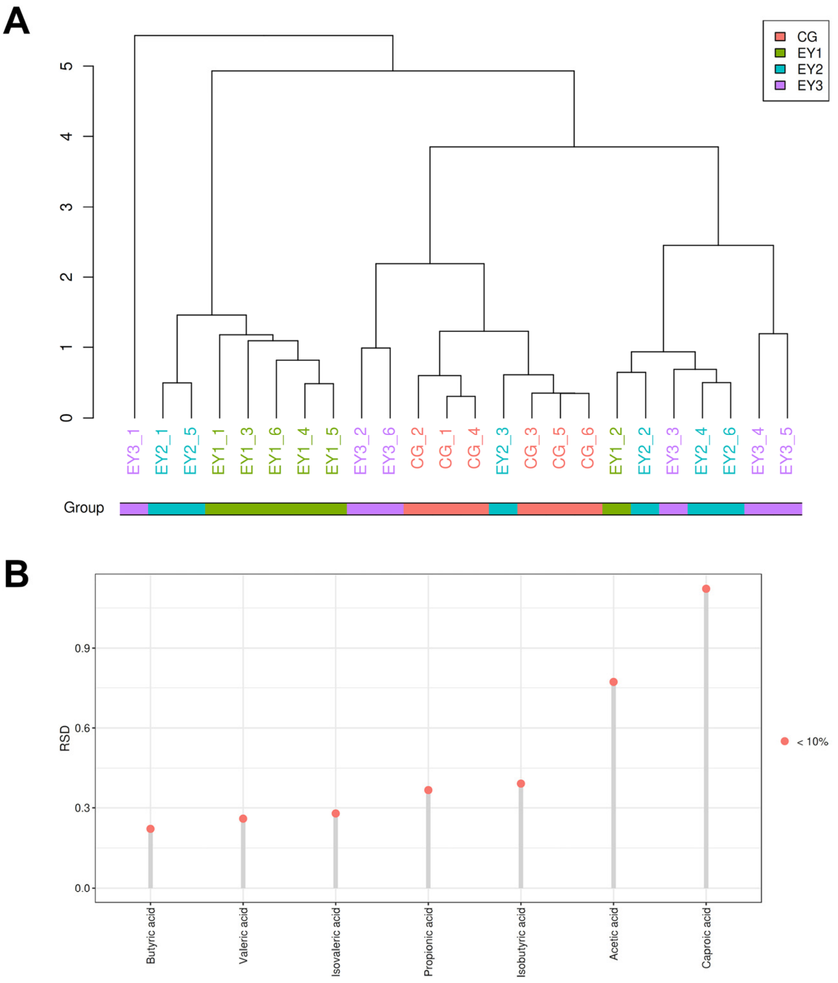

Figure S2. Data quality control and hierarchical cluster analysis ($n = 6$). (**A**) Hierarchical clustering of all samples. (**B**) Relative standard deviations (RSD) of the data. CG = the diet with a net energy level of 9.82 MJ/kg; EY1 = the diet with a net energy level of 9.57 MJ/kg; EY2 = the diet with a net energy level of 9.32 MJ/kg; EY3 = the diet with a net energy level of 9.07 MJ/kg.
